# Predictors and Prognostic Implications of Cardiac Arrhythmias in Patients Hospitalized for COVID-19

**DOI:** 10.3390/jcm10010133

**Published:** 2021-01-02

**Authors:** Maura M. Zylla, Uta Merle, Johannes A. Vey, Grigorios Korosoglou, Eva Hofmann, Michael Müller, Felix Herth, Werner Schmidt, Erwin Blessing, Christoph Göggelmann, Norbert Weidner, Mascha O. Fiedler, Markus A. Weigand, Florian Kälble, Christian Morath, Johannes Leiner, Meinhard Kieser, Hugo A. Katus, Dierk Thomas

**Affiliations:** 1 Department of Cardiology, Medical University Hospital, Im Neuenheimer Feld 410, 69120 Heidelberg, Germany; eva.hofmann@med.uni-heidelberg.de (E.H.); Christoph.Goeggelmann@med.uni-heidelberg.de (C.G.); Johannes.Leiner@med.uni-heidelberg.de (J.L.); sekretariat.katus@med.uni-heidelberg.de (H.A.K.); dierk.thomas@med.uni-heidelberg.de (D.T.); 2 German Center for Cardiovascular Research (DZHK), Partner site Heidelberg/Mannheim, University of Heidelberg, Im Neuenheimer Feld 410, 69120 Heidelberg, Germany; 3 Heidelberg Center for Heart Rhythm Disorders (HCR), Medical University Hospital Heidelberg, Im Neuenheimer Feld 410, 69120 Heidelberg, Germany; 4 Department of Gastroenterology, Hepatology and Infectious Disease, Medical University Hospital, Im Neuenheimer Feld 410, 69120 Heidelberg, Germany; uta.merle@med.uni-heidelberg.de; 5 Institute of Medical Biometry and Informatics, Im Neuenheimer Feld 130.3, 69120 Heidelberg, Germany; vey@imbi.uni-heidelberg.de (J.A.V.); kieser@imbi.uni-heidelberg.de (M.K.); 6 GRN Klinikum Weinheim, Klinik für Kardiologie, Angiologie und Pneumologie, Röntgenstraße 1, 69469 Weinheim, Germany; gkorosoglou@hotmail.com; 7 Department of Pulmonary and Respiratory Critical Care Medicine, Thoraxklinik, Heidelberg University Hospital, Röntgenstr. 1, 69126 Heidelberg, Germany; Michael.Mueller@med.uni-heidelberg.de (M.M.); Felix.Herth@med.uni-heidelberg.de (F.H.); 8 German Center for Lung Research (DZL)Translational Lung Resarch Center (TLRCH), University of Heidelberg, Im Neuenheimer Feld 156, 69120 Heidelberg, Germany; 9 Department of Anaesthesiology, Intensive Care, Pain Therapy, Thoraxklinik, Heidelberg University Hospital, Röntgenstr. 1, 69126 Heidelberg, Germany; Werner.Schmidt@med.uni-heidelberg.de; 10 SRH Klinikum Karlsbad-Langensteinbach, Abteilung für Innere Medizin, Guttmannstraße 1, 76307 Karlsbad, Germany; Erwin.Blessing@kkl.srh.de; 11 Spinal Cord Injury Center, Heidelberg University Hospital, Schlierbacher Landstraße 200a, 69118 Heidelberg, Germany; Norbert.Weidner@med.uni-heidelberg.de; 12 Department of Anaesthesiology, Heidelberg University Hospital, Im Neuenheimer Feld 110, 69120 Heidelberg, Germany; Mascha.Fiedler@med.uni-heidelberg.de (M.O.F.); Markus.Weigand@med.uni-heidelberg.de (M.A.W.); 13 Nierenzentrum Heidelberg, Department of Nephrology, Heidelberg University Hospital, Im Neuenheimer Feld 162, 69120 Heidelberg, Germany; Florian.Kaelble@med.uni-heidelberg.de (F.K.); Christian.Morath@med.uni-heidelberg.de (C.M.)

**Keywords:** arrhythmia, COVID-19, hospitalization, atrial fibrillation, risk stratification

## Abstract

Background: Cardiac manifestation of COVID-19 has been reported during the COVID pandemic. The role of cardiac arrhythmias in COVID-19 is insufficiently understood. This study assesses the incidence of cardiac arrhythmias and their prognostic implications in hospitalized COVID-19-patients. Methods: A total of 166 patients from eight centers who were hospitalized for COVID-19 from 03/2020–06/2020 were included. Medical records were systematically analyzed for baseline characteristics, biomarkers, cardiac arrhythmias and clinical outcome parameters related to the index hospitalization. Predisposing risk factors for arrhythmias were identified. Furthermore, the influence of arrhythmia on the course of disease and related outcomes was assessed using univariate and multiple regression analyses. Results: Arrhythmias were detected in 20.5% of patients. Atrial fibrillation was the most common arrhythmia. Age and cardiovascular disease were predictors for new-onset arrhythmia. Arrhythmia was associated with a pronounced increase in cardiac biomarkers, prolonged hospitalization, and admission to intensive- or intermediate-care-units, mechanical ventilation and in-hospital mortality. In multiple regression analyses, incident arrhythmia was strongly associated with duration of hospitalization and mechanical ventilation. Cardiovascular disease was associated with increased mortality. Conclusions: Arrhythmia was the most common cardiac event in association with hospitalization for COVID-19. Older age and cardiovascular disease predisposed for arrhythmia during hospitalization. Whereas in-hospital mortality is affected by underlying cardiovascular conditions, arrhythmia during hospitalization for COVID-19 is independently associated with prolonged hospitalization and mechanical ventilation. Thus, incident arrhythmia may indicate a patient subgroup at risk for a severe course of disease.

## 1. Introduction

The global pandemic caused by coronavirus disease 2019 (COVID-19) continues to evolve and exerts tremendous strain on healthcare systems. Pathomechanisms differentiating patients at high risk for adverse outcomes from those displaying minor symptoms and a mild course of disease are insufficiently understood. Cardiovascular involvement and myocardial injury are commonly observed and associated prognostic implications have been suggested by early reports [1,2,3,4]. Impairment of ventricular function and inflammatory myocardial processes have been detected in COVID-19 patients [5,6]. However, evidence regarding prevalence and prognostic effects of cardiac arrhythmias in COVID-19 is sparse and mainly limited to single center observations. Early studies among the first patient cohorts analyzed in China reported an incidence of cardiac arrhythmia of 17%, with rates of up to 44% in patients admitted to ICU [7]. However, there was no information on the respective types of arrhythmia diagnosed. Another monocentric study from Pennsylvania reported similar rates of arrhythmia events during hospitalization for COVID-19, in particular in patients treated in an intensive care setting. AF was identified as the most common arrhythmic event [8]. In a cohort of 30 patients from Italy, a possible association of cardiac arrhythmias and increased inflammatory markers as well as mortality in COVID-19 was pointed out, however, with limited reliability of results due to the small sample size [9]. Insight into the role of arrhythmias in COVID-19 is of particular importance as widely used pharmacological therapies attempting to mitigate the course of disease may be associated with proarrhythmic effects [10]. This study aims to systematically investigate the prevalence of cardiac arrhythmias in patients hospitalized for COVID-19 at multiple centers, to identify risk factors for the occurrence of cardiac arrhythmias, and to assess their role regarding clinical course and patient prognosis.

## 2. Methods

### 2.1. Patient Selection

All patients with COVID-19 who had been hospitalized at the participating centers were included in this registry from 5 March to 17 June 2020. Only patients with a complete documentation of the clinical stay (i.e., discharged from the respective center or deceased) were considered. Further inclusion criteria were a confirmation of SARS-CoV-2 infection by polymerase chain reaction testing of a nasopharyngeal sample and a duration of hospital stay for ≥ 24 h. In total, 166 patients with were included in this registry. In the majority of patients, COVID-19 was the reason for hospitalization. A subgroup of nine patients had been admitted for different indications and SARS-CoV-2-infection was diagnosed and treated in the course of the hospital stay. The study was conducted in accordance with the Declaration of Helsinki and approved by the ethics committee of the University of Heidelberg (study registration number at the ethics committee: S-281/2020). Due to the retrospective, non-interventional nature of the study based solely on data generated and documented during clinical routine processes, informed and written consent was not required in accordance with the statement of the local ethics committee.

### 2.2. Data Collection

All available clinical records including physicians’ and nurses’ written reports, diagnostic test results, telemetry logs and electronic patient files were used for systematic data collection. Demographic and clinical baseline parameters, comorbidities, biomarkers, medical therapy as well as information on endpoints regarding the clinical course of disease were extracted. Clinically relevant arrhythmias previously diagnosed or incident during hospitalization were recorded and classified according to arrhythmia diagnosis. Electrocardiograms (ECGs) conducted at admission to hospital were evaluated regarding QRS and QTc duration, if available. If ECG documentation of the arrhythmia was available in the patient file, the diagnosis was confirmed by electrophysiology experts of the study group. With respect to biomarker analyses, peak values of high-sensitive troponin T (hsTnT), N-terminal pro-B-type natriuretic peptide (NTproBNP), lactate dehydrogenase (LDH), C-reactive protein (CRP) and interleukin-6 (IL-6) were recorded, if assessed during hospitalization. Data on body mass index (BMI) or presence of obesity were available in 138 patients. Obesity was defined as BMI > 25 kg/m^2^ for the purpose of this study.

### 2.3. Statistical Methods

The patient cohort was described using summary measures of the empirical distribution. Continuous variables are reported as median (with inter-quartile range, 25th percentile = P_25_; 75th percentile = P_75_) or mean ± standard deviation (SD). The *t*-test or Mann-Whitney-Wilcoxon-test were applied for between-group comparisons. Dichotomous variables are presented as absolute and relative frequencies and were compared applying the Fisher Boschloo-test from the R package “Exact” [11].

For the purpose of selecting variables with predictive impact on the incidence of arrhythmia, the variables sex, age, hypertension, cardiovascular disease, hydroxychloroquine, and combined therapy with hydroxychloroquine and azithromycin were initially considered in terms of variable selection. First, regularized logistic regression using the elastic net penalty implemented in the package “glmnet” was computed [12,13]. The hyperparameters α (elastic net mixing parameter) and β (shrinkage parameter) were tuned conducting 5-fold cross-validation (CV) and a grid search. Subsequently, multiple logistic regression modeling was conducted only incorporating the selected variables, to estimate the odds ratios (ORs) and their 95% confidence intervals (CI). The area under the curve (AUC) value was computed applying the receiver operating characteristics (ROC) curve to evaluate the model using the package “pROC” [14]. To prevent overestimation of the model’s performance measure, the AUC-value was calculated applying 5-fold CV. During 5-fold CV, each patient is part of the training set for four times and is assigned exactly once to the testing set. Hence, in each step a model is fitted based on 80% of the data whereas a probability of the remaining 20% of the patients is estimated with respect to the incidence of arrhythmia.

Information on left ventricular ejection fraction (LVEF) was only available in 44 patients. To account for a potential influence of LVEF on the development of cardiac arrhythmia, we performed attempts to impute the missing data (Supplementary Materials). Due to a high number of missing values, LVEF was omitted from further analyses to ensure reliability of the data.

To evaluate the impact of biomarkers on the incidence of arrhythmia, univariate logistic regression modeling was performed. The AUC-values und the Youden index for identifying the optimal cut-off value were computed for each biomarker, respectively [15]. Confidence intervals of the AUC-values were calculated according to DeLong [16].

To assess the prognostic impact of arrhythmia on clinical outcomes univariate and multiple regression modeling was performed. To preserve the validity of multiple regression modeling in the light of the limited number of patients, the models were adjusted for a maximum of two additional covariates. Age and cardiovascular disease were chosen due to their clinical significance regarding outcome in COVID-19 shown by previous studies [2,17].

Due to the high proportion of newly diagnosed atrial fibrillation (AF), we conducted a subgroup analysis comparing patients with incident AF to patients who neither had a history of AF nor displayed AF in the course of the hospitalization. Due to the small sample size in the subgroup of patients with incident AF, only descriptive analyses were performed.

Due to the exploratory character of this analysis, the *p*-values are interpreted only in a descriptive sense and no adjustment for multiple testing was applied [18]. *p*-values < 0.05 were denoted as statistically significant. The statistical analysis was performed using R version 4.0.2. [19] and SPSS version 25.

## 3. Results

### 3.1. Baseline Characteristics and Outcomes in Overall Cohort

Median age was 64.1 ± 16.7 years, and the majority of patients were male (*n* = 108, 65.1%). Arterial hypertension constituted the most common risk factor (*n* = 83, 50.0%). Cardiovascular disease was present in 18.1% (*n* = 30), cardiomyopathy in 3.0% (*n* = 5), diabetes mellitus in 17.5% (*n* = 29), and obesity in 21.7% (*n* = 36). Other relevant comorbidities comprised pulmonary disease (e.g., chronic obstructive pulmonary disease, pulmonary fibrosis) in 14.5% (*n* = 24), previous or active cancer disease in 8.4% (*n* = 14), and conditions associated with immunodeficiency (e.g., due to chronic immunosuppression therapy after organ transplantation or hematological disease) in 8.4% (*n* = 14). Information on left ventricular ejection fraction (LVEF) was available in 44 patients (26.5%). Mean LVEF in this subgroup was 53.0 ± 12.3%. In three patients a cardiac pacemaker had been implanted (1.8%). Implanted cardioverter-defibrillators (ICDs) were present in two patients (1.2%). Seven patients (4.2%) presented with syncope in association with COVID-19 prior to hospital admission.

**Figure 1:**
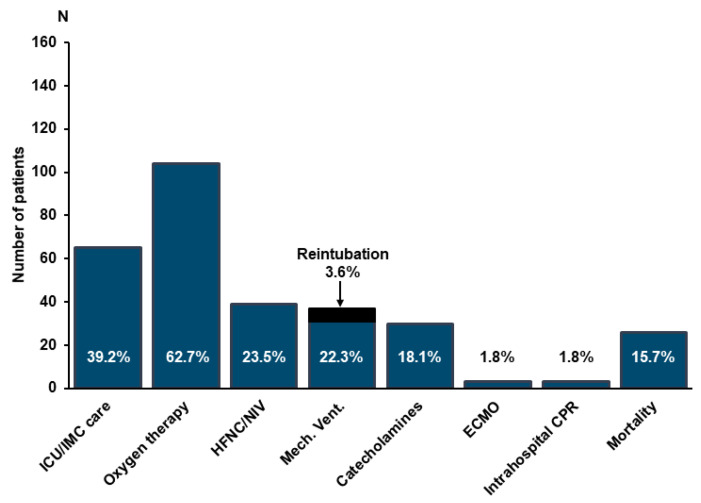
Clinical outcome in the overall cohort. Clinical endpoints are depicted as number of cases and percentage in relation to the entire cohort of 166 patients. CPR = cardiopulmonary resuscitation; ECMO = extracorporal membrane oxygenation; HFNC = high flow nasal canula; ICU = intensive care unit; IMC = intermediate care; NIV = non-invasive ventilation.

Median duration of hospitalization was 10.5 days (P_25_: 5 days; P_75_: 22 days, *n* = 154). In 12 patients, data on duration of hospitalization could not be assessed due to transfer to a different center not participating in this study. The majority of patients required oxygen therapy in the course of hospital stay, and over a third of all patients was admitted to intensive care (ICU) or intermediate care units (IMC) (Figure 1). Median duration of ICU/IMC-therapy was 8 days (P_25_: 4 days; P_75_: 22.5 days). High-flow oxygen therapy and/or non-invasive ventilation (NIV) by continuous positive airway pressure (CPAP) was necessary in 39 patients (23.5%), and pharmacological circulatory support by vasopressors was provided in 30 patients (18.1%). Thirty-seven patients (22.3%) received mechanical ventilation with a median duration of 17 days (P_25_: 7.5 days; P_75_: 26 days). Of these, six patients had to be re-intubated after initially successful weaning (Figure 1). Only a minority of patients underwent extracorporeal membrane oxygenation (ECMO) or in-hospital cardiopulmonary resuscitation (Figure 1).

With respect to cardiovascular events, myocarditis was suspected in one patient based on cardiac biomarker-kinetics and mildly reduced LVEF in echocardiography. This patient died due to respiratory failure during the hospital stay. However, the diagnosis of myocarditis could not be confirmed upon autopsy. Four patients were diagnosed with myocardial infarction (2.4%) during hospitalization. Percutaneous coronary intervention (PCI) was performed in two cases, of which one patient presented with ST segment elevation myocardial infarction (STEMI) and one patient with non-ST segment elevation myocardial infarction (NSTEMI). In the other two cases diagnosed with NSTEMI, medical therapy alone was preferred due to clinical instability with predominant respiratory symptoms and stable echocardiographic and ECG findings. One patient with NSTEMI and PCI died in the course of hospitalization due to mesenteric ischemia. Stroke or transient ischemic attack (TIA) was seen in three patients (1.8%). Twenty-six patients died during hospitalization, predominantly due to respiratory failure (*n* = 20, 76.9%) or other non-cardiac reasons (*n* = 5, 19.2%). Only one death was attributed to cardiac causes (3.8%), i.e., cardiac circulatory failure in a mechanically ventilated patient with pre-existent severe cardiovascular disease.

### 3.2. Arrhythmias During Hospitalization for COVID-19

In our cohort 34 patients (20.5%) displayed arrhythmias during hospitalization. In 17 cases (10.2%), the arrhythmia type occurring during hospital stay had already been previously diagnosed in the respective patients prior to SARS-CoV-2 infection. Specifically, in 16 patients previously diagnosed AF occurred, in one patient previously observed bradycardia was recorded. Twenty-two patients (13.3%) displayed new-onset arrhythmia that had not been diagnosed before-either without any previous arrhythmia history or in addition to other previously diagnosed arrhythmia. Of these, 16 patients (9.6%) had never been diagnosed with any type of arrhythmia prior to hospitalization for COVID-19.

**Figure 2:**
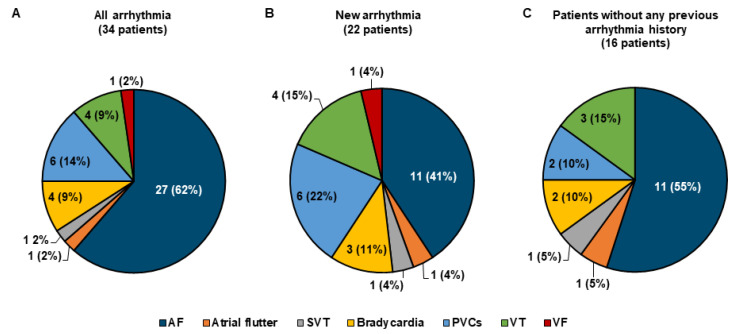
Arrhythmia diagnoses during hospitalization for COVID-19. (**A**) Distribution of arrhythmia types in all patients with arrhythmia during hospitalization. (**B**) Newly-diagnosed arrhythmia types during hospitalization. (**C**) Newly-diagnosed arrhythmia types in the subgroup of patients without any previous history of arrhythmia. Numbers depicted in the diagram reflect number and proportion of arrhythmia diagnoses. As one patient may have had multiple arrhythmia types, this number does not correspond to group size of patients. AF = atrial fibrillation; PVC = premature ventricular complexes; SVT = supraventricular tachycardia; VF = ventricular fibrillation; VT = ventricular tachycardia.

With regard to arrhythmia diagnosis during hospitalization for COVID-19, AF was the most common type of arrhythmia recorded (Figure 2A). Similarly, in patients diagnosed with a new arrhythmia type during hospitalization (Figure 2B) and in patients without any previous arrhythmia diagnosis prior to hospitalization (Figure 2C), AF constituted the most common incident arrhythmia. Bradycardia was recorded in four cases, of which one patient already had previously documented asymptomatic bradycardia. No pacemaker implantation was necessary in this subgroup. In addition, patients with frequent PVCs and ventricular tachycardia (VT) constituted relevant subgroups. All recorded VT-episodes were non-sustained. Ventricular fibrillation occurred in one patient with cardiovascular disease who had previously received an ICD due to ventricular tachycardia. In three patients (1.8%) electrical cardioversion was performed for termination of hemodynamically compromising AF. Amiodarone for pharmacological cardioversion was administered in four patients (2.4%), and chronic antiarrhythmic medical therapy was initiated in three patients (1.8%). Patients with arrhythmias more often received therapeutic anticoagulation therapy (Table 1) in accordance with the high proportion of patients with AF in this subgroup. Reasons for therapeutic anticoagulation in patients without arrhythmias were previous or new diagnosis of venous thrombosis or pulmonary embolism.

### 3.3. Clinical Predictors for New-Onset Arrhythmias

We analyzed predisposing factors associated with arrhythmias during hospitalization for COVID-19 comparing baseline characteristics of patients with arrhythmia and patients without arrhythmia during hospitalization for COVID-19 (Table 1). Patients with arrhythmias were older and more often had been diagnosed with hypertension and cardiovascular disease (Table 1). QTc duration at baseline was longer in patients with arrhythmia, however, median values were within the physiological range in both groups (Table 1). Furthermore, we analyzed the prevalence of potentially proarrhythmic medication administered in the context of COVID-19. In both subgroups, hydroxychloroquine was used in 44.1% and 44.6% of patients, respectively (Table 1). A smaller fraction of patients in both groups additionally received azithromycin. There was no statistically significant difference in the use of QT-prolonging drugs between both groups (Table 1). Median QTc-duration at baseline was within normal range in patients who later received hydroxychloroquine (409.0 ms; P_25_: 390.5 ms, P_75_: 421.5 ms).

Regularized logistic regression led to the selection of the variables age, cardiovascular disease and hypertension with respect to the prediction of arrhythmia incidence. The subsequently fitted multiple logistic regression model revealed significant association of age (OR 1.036; 95% CI 1.004–1.074; *p* = 0.036) and cardiovascular disease (OR 3.307; 95% CI 1.329–8.232; *p* = 0.01) with incident arrhythmia in COVID-19, whereas the effect of hypertension was not significant (OR 2.08; 95% CI 0.794–5.796; *p* = 0.144). As measure of the model’s performance an area under the curve (AUC) value of 0.74 (95% CI: 0.65; 0.84) was estimated by applying 5-fold cross-validation (Supplementary Figure S1). Left ventricular ejection fraction was documented in 47.1% of patients with incident arrhythmia and only 21.2% of cases without arrhythmia during hospitalization. Attempts at imputing LVEF and including the imputed dataset in the final logistic regression model hinted at a potential role of LVEF as an additional predictor for arrhythmia incidence (Table S1). However, due to the high number of missing values, LVEF was omitted from the final logistic regression model to ensure reliability of statistical analyses.

**Figure 3:**
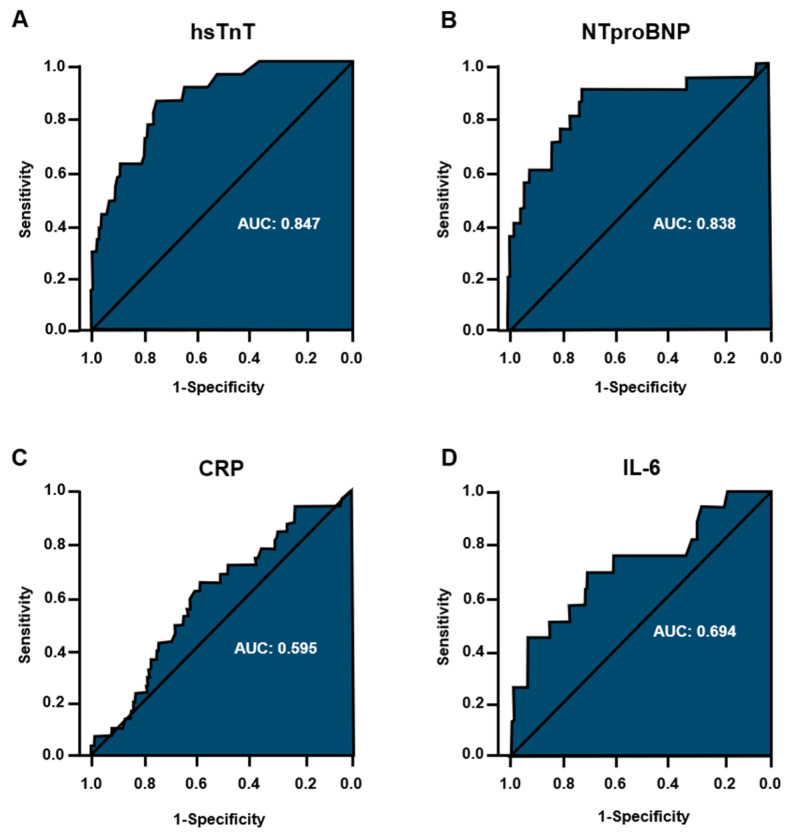
Receiver operating characteristic curve (ROC) analyses for biomarkers in relation to incident arrhythmia. ROC curves and the corresponding area under the curve (AUC) values were calculated after conducting univariate logistic regression modeling to predict incident arrhythmia for each biomarker, respectively. (**A**) hsTNT = high-sensitive troponin T; optimal cut-off value according to Youden index analysis: 27.5 pg/mL. (**B**) NTproBNP = *n*-terminal pro-B-type natriuretic peptide; cut-off value: 1518 ng/L. (**C**) CRP = C-reactive protein; cut-off value: 120.5 mg/l. (**D**) IL-6 = interleukin-6; cut-off value: 82.5 ng/L.

With regard to peak levels of cardiac and inflammatory biomarkers assessed during hospitalization, patients with arrhythmia displayed higher levels of hsTnT and NTproBNP (Figure 3A, B). Additionally, a more pronounced increase in IL-6 and LDH could be detected in the arrhythmia subgroup, whereas there was no statistically significant difference in peak levels of CRP between groups (Table 2, Figure 3C, D).

### 3.4. Prognostic Implications of Arrhythmia on Clinical Outcome

Overall duration of hospitalization was longer in patients with arrhythmia associated with COVID-19 (Table 3). Univariate analysis showed an increase of hospitalization duration of 11.4 days with the presence of incident arrhythmia (95% CI 6.05–16.7 days; *p* <0.001). Additionally, patients with arrhythmia were more often admitted to ICU or IMC wards (OR 2.37; 95% CI 1.10–5.09; *p* = 0.03), and incident arrhythmia was associated with a longer duration of hospitalization on ICU/IMC wards (Table 3). Patients with incident arrhythmia more often received vasopressors for circulatory support and non-invasive ventilation or high-flow oxygen-therapy (Table 3). Importantly, patients with arrhythmia more often presented with severe respiratory failure requiring mechanical ventilation (OR 6.69; 95% CI 2.92–15.35; *p* < 0.001). Duration of mechanical ventilation was not significantly different between patients with and without arrhythmia (Table 3).

With regard to cardiac events, myocardial infarction was more common in the patient group with arrhythmia, however, with a low overall number of events (Table 3). Stroke or transient ischemic attack (TIA) occurred in one case in the patient group with arrhythmia who had a prior diagnosis of AF and frequent PVCs, and in two patients without arrhythmia. All patients with myocardial infarction or stroke/TIA had received anticoagulation therapy with low-molecular-weight-heparin, however, in one patient heparin-therapy was paused after coronary angiography due to severe bleeding complications. In-hospital mortality was significantly elevated in COVID-19 patients with incident arrhythmia during hospitalization (OR 3.02; 95% CI 1.22–7.46; *p* = 0.02). Multiple regression analyses adjusting for differences in baseline parameters revealed that the incidence of arrhythmia constitutes a more powerful prognostic factor regarding hospitalization duration and the need for mechanical ventilation than age and prevalence of cardiovascular disease (Table 4). Finally, previous diagnosis of cardiovascular disease in our cohort was significantly associated with mortality in these analyses.

### 3.5. Subgroup Analysis of Patients with Atrial Fibrillation

Atrial fibrillation constituted the most common incident arrhythmia during hospitalization for COVID-19. In the subgroup analysis of patients with incident AF, age, hypertension and cardiovascular disease were associated with incidence of the arrhythmia (Table 5). Both cardiac and inflammatory markers showed a stronger increase in patients with AF. Similar to the effects of incident arrhythmia in the overall cohort, AF itself was associated with longer overall hospitalization times and longer duration of ICU/IMC care. Additionally, an increased need for high-flow oxygen therapy or non-invasive ventilation, mechanical ventilation and pharmacological circulatory support could be seen in this subgroup. Information regarding initiation of anticoagulation therapy was available in 10 patients with AF during hospitalization for COVID-19. In six cases, low-molecular-weight heparin (LMWH) in therapeutic doses was applied, two other patients received NOACs. Two patients only received prophylactic doses of LMWH, in one case due to a low CHA2DS2-Vasc-Score of 1 and a self-limiting AF-episode, in one case due to delayed diagnosis of AF. Two patients in the AF-subgroup were diagnosed with myocardial infarction. In both cases, anticoagulation therapy had been initiated with LMWH. One patient underwent PCI and died in the later course of the hospitalization due to mesenteric ischemia. In this patient anticoagulation therapy had been paused for 22 days after coronary angiography due to severe bleeding complications requiring transfusion therapy. The other patient received medical treatment as a type-II myocardial infarction was suspected due to stable echocardiography und ECG-findings. All cases of death in patients with AF were attributed to non-cardiac causes: in addition to the patient described above three patients deceased due to respiratory failure.

## 4. Discussion

Cardiac arrhythmias were the most common cardiac event associated with hospitalization for COVID-19. Age and cardiovascular disease were identified as risk factors for the incidence of arrhythmias. Arrhythmia was associated with elevated cardiac biomarkers suggesting myocardial injury, need for ICU/IMC care and mechanical ventilation as well as mortality, and constituted an independent predictor of prolonged hospitalization and need for mechanical ventilation.

This is the first multicenter study including both tertiary care centers and regional hospitals focusing on the role of arrhythmias in hospitalization for COVID-19, considering both clinical and biomarker profiles. With respect to age, baseline parameters and the overall incidence of arrhythmias, our cohort corresponds to previously described COVID-19 patient populations [7,8,17,20].

In accordance with prior results, AF was the most common incident arrhythmia both in the entire cohort and in the subgroup of patients without any previous history of arrhythmia. An elevated risk for AF has been described in association with other respiratory virus infections, in particular influenza [21], however, with a lower incidence compared to our cohort of COVID-19-patients [22]. The present analyses of the patient subgroup with newly diagnosed AF revealed not only a relevant increase in cardiac biomarkers hinting at myocardial injury but also significantly elevated inflammatory biomarkers. This may point to an association between the degree of inflammatory state caused by COVID-19 and susceptibility to AF, which is in line with previous findings regarding inflammatory mechanisms promoting the development of atrial fibrillation [23].

With respect to risk factors for arrhythmia incidence during hospitalization for COVID-19, we could identify age and previous cardiovascular disease as predictors for the occurrence of any arrhythmia. These baseline characteristics have previously been shown to predispose for the development of arrhythmia in the general population without association with infectious diseases [24]. Thus, they may reflect a subgroup of greater general susceptibility to additional proarrhythmic effects. In patients with arrhythmia in our cohort, QTc duration was longer at admission, albeit within normal range in the majority of patients. Dynamic changes of the QTc interval under therapy with hydroxychloroquine and azithromycin cannot be evaluated as repeated ECGs during hospital stay were not systematically available in all patients. Thus, proarrhythmic effects of these drugs cannot be excluded in this cohort. Previous reports point towards significant prolongation of the QTc interval by hydroxychloroquine in COVID-19 which is even more enhanced in combination with azithromycin therapy [25,26]. However, the rate of associated ventricular arrhythmia has been low in these studies. Similarly, no typical “*torsades des pointes*” were seen in our cohort: in three of five patients with ventricular arrhythmias pre-existent cardiovascular disease was present, and only one of the remaining two patients received QTc-prolonging medication. Thus, rather than induced by direct effects of administered medication, ventricular arrhythmias may have been due to other predisposing risk factors in our cohort.

Patients with arrhythmias during hospitalization showed elevated cardiac biomarkers and elevated levels of IL-6. Myocardial injury in COVID-19 has been reported by multiple studies, however, the underlying mechanisms have yet to be elucidated [2,4]. In our cohort, arrhythmia itself may have promoted a more pronounced increase in hsTNT and NTproBNP by an additional shift in myocardial oxygen demand under a restricted respiratory function and by increasing atrial and ventricular load. In particular, among patients with pre-existent cardiovascular disease a myocardial supply-demand-imbalance of oxygen may become evident during arrhythmia. On the other hand, myocardial injury or ischemia-especially in the light of a higher prevalence of cardiovascular disease in this subgroup-may have exerted proarrhythmic effects in addition to the inflammatory state. Such additional inflammatory influences are implicated by the pronounced increase in IL-6 in patients with arrhythmias in our cohort. In COVID-19 a state of hyperinflammation is commonly observed and correlates with both respiratory failure and myocardial injury [27]. Based on our observations, it may additionally constitute a risk factor for the incidence of arrhythmia but distinct molecular mechanisms have yet to be investigated in experimental studies and larger, prospective patient cohorts. Estimating optimal cut-off values is associated with uncertainty and is affected by the study population. Therefore, the calculated cut-offs for the biomarkers in this study should be interpreted with caution.

With respect to clinical outcome, an association of incident arrhythmia with the need for ICU/IMC-care was identified, which is in line with the previous observations from Pennsylvania [8]. However, age and cardiac co-morbidities have been identified as potential confounders in our analyses regarding ICU/IMC admission. Furthermore, rates of NIV- or high-flow oxygen-therapy and need for vasopressors were increased in this group. These observations, together with the results from biomarker analyses, reflect an association of the severity of disease with the incidence of cardia arrhythmia in our cohort.

More specifically, we show that mechanical ventilation is associated with the occurrence of arrhythmias, even when correcting for age and cardiovascular disease as potential confounders. Additionally, overall duration of hospitalization was significantly increased in patients with incident arrhythmia in multiple regression analyses, which has not been reported by previous studies. This result points towards a potentially independent prognostic role of arrhythmia in COVID-19. In light of limited ventilator- and hospital-capacity during peak episodes of the COVID-19-pandemic, these aspects are of particular relevance. In this context, our exploratory univariate analysis of patients with atrial fibrillation shows that not only ventricular arrhythmias but also supraventricular arrhythmias may have implications on the clinical course during hospitalization COVID-19 patients.

Arrhythmia during hospitalization for COVID-19 was associated with increased in-hospital mortality in the univariate analysis. However, in multiple regression analyses, pre-existent cardiovascular disease had a stronger prognostic implication than incident cardiac arrhythmia regarding this aspect, even though the majority of fatal cases were due to respiratory failure. This is in line with observations from large multicenter cohorts identifying risk factors for in-hospital death in COVID-19 [28]. With respect to a wide range of symptoms and prognostic severity associated with an infection with SARS-CoV-2, further studies aiming at individualized risk stratification are crucial.

### Limitations

Due to its retrospective design, this study carries inherent limitations. Despite thorough analysis of clinical records and use of different source documents (e.g., discharge notes, nurses’ reports, daily doctors’ documentation) underreporting of arrhythmia events cannot be excluded. Not all arrhythmic events during the clinical course may have been documented in written reports. Asymptomatic arrhythmias in patients without continuous ECG-monitoring may also have been missed. However, clinically relevant arrhythmias leading to medical interventions are documented as part of the participating centers’ standards.

Baseline and outcome data recorded in this study were prespecified and screened for in the available clinical documents. Missing parameters were specifically inquired from the participating centers. Due to different admission protocols and diagnostic standards, there are remaining missing values with regard to certain baseline parameters or biomarker measurements. However, we clearly indicate this limitation in the respective tables whenever information was available only in a subgroup of patients. QTc-duration was available at baseline in the majority of patients, however, due to different standards of ECG-based follow-up, QTc-duration in the course of hospitalization, e.g., during therapy with QT-prolonging drugs, could not be systematically analyzed.

Inclusion of both tertiary and secondary-level hospitals may lead to treatment bias due to different standards of care or available facilities. Importantly, in our study, all contributing centers provide intermediate and intensive care units and operate according to national and international guidelines. Cardiorespiratory monitoring, non-invasive and mechanical ventilation are carried out according to guidelines in all participating centers. All centers treated both moderate and severe cases of COVID-19. Patients requiring extracorporeal life support were primarily treated at tertiary centers but constituted a minority of subjects in this study cohort. Therefore, we do not expect significant bias due to differences in center size. However, due to the limited number of patients in the respective subgroups a comprehensive analysis of this aspect was not feasible. In order to provide further insight, we present an overview into the types and individual contribution of participating centers (Table S1). Additionally, individual specific therapy attemps with respect to COVID-19, e.g., hydroxochloroquine administration, were specified (Table 1). Left ventricular ejection fraction (LVEF) may have constituted an additional predictor for arrhythmia, however, the value was not provided in a relevant number of patients in this cohorts. In order to account for this limitation, we attempted imputation of these values (Supplementary Materials) hinting at a potential role of reduced LVEF as a risk factor for arrhythmia during hospitalization for COVID-19. However, these results are exploratory and have to be interpreted with caution due to the high number of missing values. Further efforts should be made to study this specific aspect in COVID-19 patients.

## 5. Conclusions

The present multicenter study identifies patients with cardiac arrhythmia during hospitalization for COVID-19 as a high-risk subgroup characterized by severe course of disease and adverse outcomes. Underlying pathomechanisms may be related to a hyperinflammatory state and myocardial injury, particularly in patients with cardiac comorbidities. Due to the high incidence of arrhythmic events in COVID-19 and their potential prognostic implications, arrhythmias should be screened for in affected patients with respective risk factors.

## Figures and Tables

**Table 1:** Comparison of baseline characteristics.

	Arrhythmia (*n* = 34)	No Arrhythmia (*n* = 132)	*p* Value *
Female	11 (32.4)	47 (35.6)	0.817
Age (years), mean ± SD	73.6 ± 12.8	61.6 ± 16.7	<0.001 ^†^
Hypertension	26 (76.5)	57 (43.2)	0.001
Cardiovascular disease	14 (41.2)	16 (12.1)	<0.001
Cardiomyopathy	1 (2.9)	4 (3.0)	1.000
LVEF (%), mean ± SD	57.1 ± 8.2 (*n* = 16)	45.7 ± 14.9 (*n* = 28)	0.002 ^†^
Diabetes	7 (20.6)	22 (16.7)	0.566
Obesity	7 (23.3) (*n* = 30)	29 (26.9) (*n* = 108)	0.789
Body mass index (kg/m^2^) median (P_25_; P_75_)	24.1 (20.9; 25.1) (*n* = 17)	23.3 (20.6; 25.7) (*n* = 80)	0.939 ^‡^
Pulmonary disease	6 (17.6)	18 (13.6)	0.566
Immunodeficiency	4 (7.6)	10 (11.8)	0.441
QRS duration (ms), median (P_25_; P_75_)	95.5 (90.0; 107) (*n* = 24)	95.0 (90.0; 105) (*n* = 107)	0.797 ^‡^
QTc duration (ms), median (P_25_; P_75_)	430 (400; 470) (*n* = 24)	407 (393; 425) (*n* = 107)	0.012 ^‡^
Hydroxychloroquine	15 (44.1)	59 (44.6)	1.000
Hydroxychloroquine + azithromycin	7 (20.6)	20 (15.2)	0.424
Anticoagulation therapy	24 (80.0) (*n* = 30)	27 (23.3) (*n* = 116)	<0.001
- LMWH/UFH	15 (50.0)	17 (14.7)	<0.001
- Oral anticoagulants	9 (30.0)	10 (8.6)	0.005
Thromboprophylaxis with LMWH/UFH	5 (16.7) (*n* = 30)	52 (44.8) (*n* = 116)	0.004

LVEF = left ventricular ejection fraction. Data are presented as count and percentage within subgroup, unless stated otherwise. * = Fisher-Boschloo-test was used unless stated otherwise. ^†^ = *t*-test. ^‡^ = Wilcoxon-Mann-Whitney-test.

**Table 2:** Peak levels of cardiac and inflammatory biomarkers.

	Arrhythmia (*n* = 34)	No Arrhythmia (*n* = 132)	*p*-Value *
hsTnT (pg/mL)	85.0 (32.0; 377) (*n* = 21)	13.0 (7.0; 30.0) (*n* = 115)	<0.001
NTproBNP (ng/L)	11,661 (3000; 33,259) (*n* = 20)	557 (187; 2346) (*n* = 101)	<0.001
CRP (mg/L)	137 (66.0; 191) (*n* = 31)	101 (44.0; 164) (*n* = 130)	0.102
Interleukin-6 (ng/L)	173 (51.1; 739) (*n* = 16)	50.0 (23.6; 123) (*n* = 92)	0.014
LDH (U/L)	514 (329; 774) (*n* = 28)	419 (323; 530) (*n* = 107)	0.012

CRP = C-reactive protein; hsTnT = high-sensitive troponin T; LDH = lactate dehydrogenase; NTproBNP = N-terminal pro-B-type natriuretic peptide. Data are presented as median and percentiles (P_25_; P_75_). * = Wilcoxon-Mann-Whitney-test.

**Table 3:** In-hospital events and clinical outcome.

	Arrhythmia (*n* = 34)	No Arrhythmia (*n* = 132)	*p*-Value *
Duration of hospitalization (days), median (P_25_; P_75_)	24.0 (10.0; 33.0) (*n* = 30)	9.0 (5.0; 15.0) (*n* = 124)	<0.001 ^†^
Admission to ICU/IMC	19 (55.9)	46 (24.8)	0.025
Duration of ICU/IMC-stay (days), median (P_25_; P_75_)	23.0 (8.0; 33.0)	7.0 (3.0; 14.5)	0.004 ^†^
Oxygen therapy	24 (70.6)	80 (60.6)	0.299
HFNC/NIV therapy	14 (41.2)	25 (19.1)	0.010
Mechanical ventilation	18 (52.9)	19 (14.4)	<0.001
Duration of mechanical ventilation (days), median (P_25_; P_75_)	17.5 (10.0; 32.5)	15.0 (7.0; 25.0)	0.599 ^†^
Vasopressor therapy	16 (47.1)	14 (10.6)	<0.001
Myocardial infarction	3 (8.8)	1 (0.8)	0.021
Death	10 (29.4)	16 (12.1)	0.026

HFNC = high-flow nasal canula; ICU = intensive care unit; IMC = intermediate care unit; NIV = non-invasive ventilation. * = Fisher-Boschloo-test was used, unless stated otherwise. ^†^ = Wilcoxon-Mann-Whitney-test.

**Table 4:** Multiple regression models of clinical outcome parameters associated with arrhythmia.

	Beta Coefficient * (95% CI)		***p*-Value**
Hospitalization Duration in Days			
Arrhythmia	8.02	(2.28; 13.8)	0.006
Cardiovascular disease	3.11	(−2.65; 8.88)	0.288
Age	0.17	(0.04; 0.30)	0.011
	Odds Ratio * (95% CI)		***p*-Value**
Admission to ICU/IMC			
Arrhythmia	1.90	(0.83; 4.38)	0.127
Cardiovascular disease	1.20	(0.50; 2.85)	0.673
Age	1.02	(0.99; 1.04)	0.161
Mechanical ventilation			
Arrhythmia	6.05	(2.46; 15.3)	<0.001
Cardiovascular disease	1.84	(0.67; 4.80)	0.222
Age	1.00	(0.97; 1.02)	0.716
Death			
Arrhythmia	1.45	(0.49; 4.01)	0.483
Cardiovascular disease	3.96	(1.45; 10.7)	0.007
Age	1.03	(1.00; 1.07)	0.066

ICU = intensive care unit; IMC = intermediate care unit. * = For continuous outcomes multiple linear regression was applied and the beta coefficients with its 95% confidence intervals (95% CI) are given, whereas dichotomous outcomes examined conducting multiple logistic regression and reporting the odds ratios (OR) with its 95% CI.

**Table 5:** Patients with incident atrial fibrillation in COVID-19.

	Atrial Fibrillation (*n* = 11)	No Atrial Fibrillation (*n* = 128)	*p*-Value
Female	2 (18.2)	45 (35.2)	0.287 *
Age (years), mean ± SD	71.6 ± 8.9	60.2 ± 16.4	0.024 ^†^
Hypertension	6 (54.5)	54 (42.2)	0.491 *
Cardiovascular disease	4 (36.4)	13 (10.2)	0.023 *
LVEF (%), mean ± SD	56.8 ± 10.1 (*n* = 5)	57.0 ± 6.9 (*n* = 24)	0.966 ^†^
Diabetes	3 (27.3)	23 (18.0)	0.358 *
Obesity	2 (22.2) (*n* = 9)	29 (26.9) (*n* = 104)	1.000 *
hsTnT (pg/mL)	132 (37.0; 993) (*n* = 7)	12.0 (7.0; 30.0) (*n* = 111)	<0.001 ^‡^
NTproBNP (ng/L)	7,304 (4,336; 32,627) (*n* = 7)	535 (187; 2358) (*n* = 99)	0.001 ^‡^
CRP (mg/L)	214 (150; 288) (*n* = 9)	103 (48.0; 164) (*n* = 126)	0.012 ^‡^
Interleukin-6 (ng/L)	2,132 (358; 4538) (*n* = 4)	50.0 (25.0; 113) (*n* = 91)	0.004 ^‡^
LDH (U/L)	719 (493; 836) (*n* = 9)	415 (319; 530) (*n* = 123)	0.003 ^‡^
Duration of hospitalization (days), median (P_25_; P_75_)	31.0 (16.0; 35.5) (*n* = 8)	9.0 (5.0; 15.0) (*n* = 120)	0.007 ^‡^
Admission to ICU/IMC	8 (72.7)	46 (35.9)	0.016 *
Duration of ICU/IMC stay (days), median (P_25_; P_75_)	28.5 (14.5; 33.0)	7.5 (3.0; 18.5)	0.019 ^‡^
Oxygen therapy	7 (63.6)	79 (61.7)	1.000 *
HFNC/NIV therapy	6 (54.5)	23 (18.1)	0.008 *
Mechanical ventilation	9 (81.8)	20 (15.6)	<0.001 *
Duration of ventilation (days), median (P_25_; P_75_)	18.0 (9.5; 33.0)	16.5 (7.3; 26.5)	0.562 ^‡^
Vasopressor therapy	7 (63.6)	15 (11.7)	<0.001 *
Myocardial infarction	2 (18.2)	2 (1.6)	0.026 *
Death	4 (36.4)	14 (10.9)	0.029 *

CRP = C-reactive protein; HFNC = high-flow nasal canula; hsTnT = high-sensitive troponin T; ICU = intensive care unit; IMC = intermediate care unit; LDH = lactate dehydrogenase; LVEF = left ventricular ejection fraction; NIV = non-invasive ventilation; NTproBNP = *n*-terminal pro-B-type natriuretic peptide. * = Fisher-Boschloo-test; ^†^ = *t*-test; ^‡^ = Wilcoxon-Mann-Whitney-test.

## Data Availability

Data including or providing clues to patient identity cannot be shared publicly because of patient confidentiality and data protection regulations according to the study protocol approved by the ethics committee. However, data used for this research work can be shared in a pseudonymized manner and are available from the corresponding author for researchers who meet the criteria for access to confidential data.

## Supplementary Materials

The following are available online at https://www.mdpi.com/2077-0383/10/1/133/s1, Figure S1: ROC curve for predicting arrhythmia, Table S1: Patient recruitment according to participating centers, Table S2: Descriptive analyses after imputation of LVEF.
