# Supplementary material for: Predictors and Prognostic Implications of Cardiac Arrhythmias in Patients Hospitalized for COVID-19
Source: J Clin Med. 2021 Jan 2;10(1):133. doi: 10.3390/jcm10010133 (PMC7796041; doi:10.3390/jcm10010133)
Supplement: Supplementary file 1 [file jcm-10-00133-s001.pdf]

Supplement to: Predictors and prognostic implications of cardiac arrhythmias in patients hospitalized for COVID-19

Short Title: Prognostic implications of arrhythmias in COVID-19

Maura M. Zylla, MD,[1,2,3] Uta Merle, MD,[4] Johannes A. Vey, M.Sc.,[5] Grigorios Korosoglou, MD,[6] Eva Hofmann, MD,[1,7] Michael Müller, MD,[7,8] Felix Herth, MD,[7,8] Werner Schmidt, MD,[9] Erwin Blessing, MD,[10] Christoph Göggelmann, MD,[1,11] Norbert Weidner, MD,[11] Mascha O. Fiedler, MD,[12] Markus A. Weigand, MD,[12] Florian Kälble, MD,[13] Christian Morath, MD,[13] Johannes Leiner,[1,3] Meinhard Kieser, PhD,[5] Hugo A. Katus, MD,[1,2,3] Dierk Thomas, MD[1,2,3]

[1]Department of Cardiology, Medical University Hospital, Im Neuenheimer Feld 410, 69120 Heidelberg, Germany;

[2]DZHK (German Center for Cardiovascular Research), partner site Heidelberg/Mannheim, University of Heidelberg, Im Neuenheimer Feld 410, 69120 Heidelberg, Germany;

[3]HCR (Heidelberg Center for Heart Rhythm Disorders), Medical University Hospital Heidelberg, Im Neuenheimer Feld 410, 69120 Heidelberg, Germany;

[4]Department of Gastroenterology, Hepatology and Infectious Disease, Medical University Hospital, Im Neuenheimer Feld 410, 69120 Heidelberg, Germany;

[5]Institute of Medical Biometry and Informatics, Im Neuenheimer Feld 130.3, 69120 Heidelberg, Germany;

[6]GRN Klinikum Weinheim, Klinik für Kardiologie, Angiologie und Pneumologie, Röntgenstraße 1, 69469 Weinheim, Germany;

[7]Thoraxklinik, Heidelberg University Hospital, Department of Pulmonary and Respiratory Critical Care Medicine, Röntgenstr. 1, 69126 Heidelberg, Germany;

[8]DZL (German Center for Lung Research), Translational Lung Resarch Center (TLRCH), University of Heidelberg, Im Neuenheimer Feld 156, 69120 Heidelberg, Germany;

[9]Thoraxklinik, Heidelberg University Hospital, Department of Anaesthesiology, Intensive Care, Pain Therapy, Röntgenstr. 1, 69126 Heidelberg, Germany;

[10]SRH Klinikum Karlsbad-Langensteinbach, Abteilung für Innere Medizin, Guttmannstraße 1, 76307 Karlsbad, Germany;

[11]Spinal Cord Injury Center, Heidelberg University Hospital, Schlierbacher Landstraße 200a, 69118 Heidelberg, Germany;

[12]Department of Anaesthesiology, Heidelberg University Hospital, Im Neuenheimer Feld 110, 69120 Heidelberg, Germany;

[13]Nierenzentrum Heidelberg, Department of Nephrology, Heidelberg University Hospital, Im Neuenheimer Feld 162, 69120 Heidelberg, Germany;

Supplemental methods

Imputation of LVEF

In the majority of patients, information on left ventricular ejection fraction (LVEF) was not provided in the source documents. Left ventricular ejection fraction was documented in 47.1% of patients with incident arrhythmia and only 21.1% of cases without arrhythmia during hospitalization. To account for a potential influence of LVEF on the incidence of cardiac arrhythmia, we performed attempts to impute the missing data. Two methods were applied to impute the variable LVEF: mean imputation and multiple imputation applying Bayesian linear regression with 10 iterations using all variables as predictors. Replacing missing values by the LVEF mean leads to the situation that each patient irrespective of the group receives the same value and thereby, no additional information is added. LVEF between the groups is still significant, when no additional information is added. Furthermore, the difference remains significant after conducting multiple imputation (Table S1).

Supplemental Table S1 Patient recruitment according to participating centers

	Type of center	Number of patients contributed
University Hospital Heidelberg (Departments of Gastroenterology, Cardiology, Anaesthesiology, Nephrology/Nierenzentrum)	Tertiary-level university hospital	99
GRN Klinikum Weinheim	Regional, secondary-level hospital	30
Thoraxklinik, University Hospital Heidelberg	Tertiary (specialized on thoracic and pulmonary disease, associated with the University Hospital Heidelberg)	17
SRH Klinikum Karlsbad-Langensteinbach	Regional, secondary-level hospital	11
Heidelberg University Orthopedic Hospital	Tertiary (specialized on orthopedic care, associated with the University Hospital Heidelberg)	9

Supplemental Table S2 Descriptive analyses after imputation of LVEF

	Total (n=166)	Arrhythmia (n=34	No arrhythmia (n=132)	P value*
**Mean imputed LVEF**				
Mean ± SD	53.0±6.3	49.6±10.7	53.9±4.1	<0.001
**Multiply imputed LVEF**				
Mean ± SD	54.9±7.6	46.3±11.2	57.1±4.2	<0.001

*= t-test

SUPPLEMENTAL FIGURE AND LEGEND

Supplemental Figure

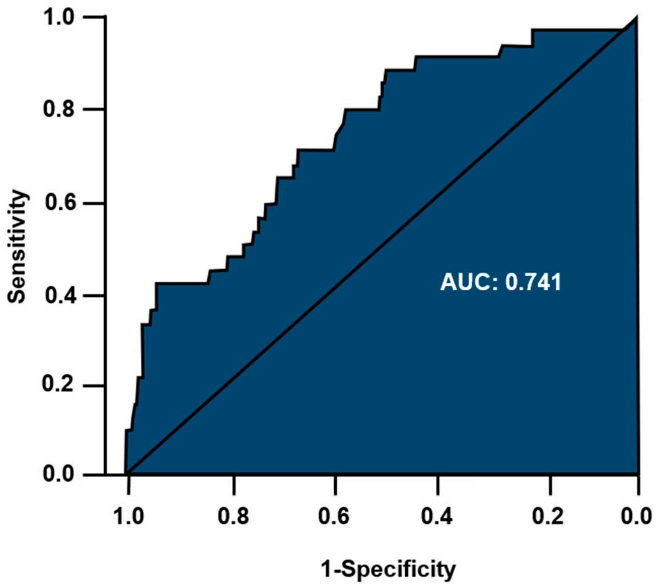

Supplemental Figure ROC curve for predicting arrhythmia

The ROC curve and the corresponding area under the curve (AUC) value was calculated for the logistic regression model including the selected variables age, hypertension and cardiovascular disease with respect to predict incident arrhythmia.
